# Supplementary material for: Chromothripsis during telomere crisis is independent of NHEJ, and consistent with a replicative origin
Source: Genome Res. 2019 May;29(5):737–49. doi: 10.1101/gr.240705.118 (PMC6499312; doi:10.1101/gr.240705.118)
Supplement: Supplemental Material [file supp_gr.240705.118_Supplemental_file_1.zip › contigs/annotated_contigs/DB50/contig.2.DB50_length_871_mean_cov_6.71641791045.docx]

**DB50_length_871_mean_cov_6.71641791045**

```
TCATCTGGGTATTAAAAAAGAGATTCCTGGACAGTTTGGCTCCAGACCTTGAGCTAGTATTTGGAAAACCCTGGAAGAGCATTCCAGGC
 >chrX:49866442-49866852 + E=1e-234
TTCATCTCACCCTAAAGTCATATCAGCCTCCTGTGTGTGGGCACCATCTTAACACCTGCCACCACCTGAGAGAACTCCTTGTCCTTCTG

CTTGTTCACTATTGTATCCCAAGCATCAAGTGTAGTGCCTAGCACAGAGCAGGACCTCAGAGAATACAGTGCTTGCTGAACACTGGCTG

GTGGTGCAAAGCTGGACTGCTTAGTTCCTGTTTCCTGCCTGGAGATATGTCTCTTACCTGGTTGTATACCACCTTCTGGGCACACTACA

CTTACCCGGCCACAGGGGGTTCTGGCTCTTACATTACAAGTCACAACCTGATCA|CAGGGTTGTAGT|AAAGTTCCTTGTACCCCATCC
                                                                   >chrX:49867853-49868
GCCCTCACCTTGTCCTCAAGCATGATGCCTCTTGCTAGTTCTAGTTACAAAATAAAAACTCCCTTGTCTATAAGTGGATACCAACAGAT
302 + E=3e-255
ATGACTGTGTGTATGTGGAAATGAGGGTTGTCTTCATTAGCAATAATATACCAGAAGTGCTTTATTACACAAACATCCTTGAACTTTAC

TACAACCCTGTGATATAGGCACAATATGAAGAACCCAAGACTTGGTTGTGATTTGCTCAGTAACTCAGAGGCAGGAAGTGATGTAACAG

AAACTGGAACTCAATGTCTTTGACCCCAAGCCCAATGATCTACTACTAATTTTGTGGCTCAGTGCTCCTGTTCATCCTGAACATTTTCA

GGCCAATTTATGGGAAGAAGGTAACAGAAGCTTGCCTGGTGAAGTTGGGCAGTTGGCCTGGTGTTAAAAATT
```
